# Vitamin D3 Inhibits *Helicobacter pylori* Infection by Activating the VitD3/VDR-CAMP Pathway in Mice

**DOI:** 10.3389/fcimb.2020.566730

**Published:** 2020-10-23

**Authors:** Anni Zhou, Lei Li, Guiping Zhao, Li Min, Si Liu, Shengtao Zhu, Qingdong Guo, Chunjie Liu, Shutian Zhang, Peng Li

**Affiliations:** ^1^ Beijing Key Laboratory for Precancerous Lesion of Digestive Disease, Department of Gastroenterology, National Clinical Research Center for Digestive Disease, Beijing Digestive Disease Center, Beijing Friendship Hospital, Capital Medical University, Beijing, China; ^2^ Department of Digestive Diseases, Affiliated Hospital for Wei Fang Medical University, Weifang, China; ^3^ Institute of Biomedical Engineering, Academy of Military Medical Sciences of the Chinese PLA, Beijing, China

**Keywords:** *Helicobacter pylori*, Vitamin D3, VitD receptor, CAMP, gastric cancer

## Abstract

*Helicobacter pylori* (*H. pylori*) infection is closely associated with the occurrence and development of gastric diseases. Therefore, eliminating *H. pylori* infection should help to prevent gastric diseases. Vitamin D3 (VitD3, 1,25(OH)_2_D_3_) was previously observed to exhibit anti-*H. pylori* infection activity in clinic, but these results were reported in heterogeneous *in vivo* studies without elucidation of the underlying mechanisms. In the present study, we established *H. pylori* infection models in both wild-type and VDR knockdown (VDR^-KD^) mice, which were used to demonstrate that VitD3 inhibits *H. pylori* infection by enhancing the expression of VitD receptor (VDR) and cathelicidin antimicrobial peptide (CAMP). Furthermore, VDR^-KD^ mice that exhibited lower VDR expression were more susceptible to *H. pylori* infection. In cultured mouse primary gastric epithelial cells, we further demonstrated that the VitD3/VDR complex binds to the CAMP promoter region to increase its expression. These data provide a mechanistic explanation of the anti-*H. pylori* infection activity of VitD3 at the molecular level in mice and suggest a new avenue for the clinical management of *H. pylori* eradication therapy.

## Introduction

Gastric diseases, including gastritis, gastric ulcers, and gastric cancer, are a serious threat to human health. According to a previous report (Bray et al., 2018), the incidence of gastric disease has markedly increased in recent years. For example, according to the latest Global Cancer Statistics 2018 reports, the incidence and mortality associated with gastric cancer, which involves the development of malignant tumors, rank fifth and third worldwide, respectively, among all cancers. However, the pathogenesis of gastric disease is complex and remains largely unclear. Among potential etiologic agents, *Helicobacter pylori* (*H. pylori*) infection is most often linked to the occurrence and development of gastric diseases (Tsukamoto et al., 2013).

*H. pylori* is a spiral-shaped, gram-negative microaerobic bacterial pathogen that infects approximately half of the world’s population (Marshall and Warren, 1984; Suerbaum and Josenhans, 2007). Patients with *H. pylori* infection have chronic active gastritis, approximately 10–20% of whom can develop peptic ulcers, while 50% develop gastric mucosal atrophy, and 1–3% may even develop gastric adenocarcinoma or gastric mucosa-associated lymphoid tissue lymphoma (Malfertheiner et al., 2017; Noto and Peek, 2017). The World Health Organization (WHO) therefore lists *H. pylori* as a Class I carcinogen, and the eradication of *H. pylori* has become an important issue in the prevention of gastric cancer (Bouvard et al., 2009; IARC working group on the evaluation of carcinogenic risks to humans: some industrial chemicals. Lyon, 15-22 February 1994, 1994). The most commonly recommended *H. pylori* eradication regimen in clinics is quadruple therapy with proton pump inhibitors, bismuth, and two antibiotics, which initially achieved good therapeutic effect. However, the widespread application of antibiotics has resulted in the emergence of drug-resistant strains of *H. pylori*, leading to a gradual decline in the effectiveness of *H. pylori* eradication (Malfertheiner et al., 2012). Vaccines are known to be an effective measure for preventing infectious diseases, and concerted efforts have been made to develop *H. pylori* vaccines in recent years, although a safe and effective vaccine is not yet available (Sutton and Lee, 2000; Malfertheiner et al., 2008; Czinn and Blanchard, 2011; Zeng et al., 2015). Exploring new strategies for the prevention and treatment of *H. pylori* infection is therefore an urgent public health priority.

Vitamin D is endogenously produced in the skin from 7-dehydrocholesterol by the action of ultraviolet light. Subsequently, Vitamin D forms 25-hydroxyvitamin D_3_ (25(OH)D_3_) and 1,25-(OH)_2_D_3_ under the action of 25-hydroxylase in hepatocyte microsomes and 1-α hydroxylase in the mitochondria of proximal tubular epithelial cells (DeLuca, 1974). 1,25(OH)_2_D_3_ (VitD3, the active form of VitD) is a fat-soluble steroid that is essential for many physiological processes in the human body, one of which is to promote the absorption of calcium and phosphorus by small intestinal mucosal cells.

In recent years, nonclassical effects of VitD3 have been described. For instance, VitD3 and its metabolites were reported to have immune regulatory functions and the capacity to promote the growth and differentiation of skin cells (Liu et al., 2006; Zasloff, 2006). Immune cells contain all the machinery required to synthesize and respond to VitD3, and this machinery is enhanced by challenge to the immune system (Gombart, 2009). Notably, VitD3 has been shown to support the clearance of *Pseudomonas aeruginosa* (*P. aeruginosa*) by use of macrophages (Nouari et al., 2016; Andrade et al., 2018) and to exert direct bactericidal activity against *Streptococcus* *mutants* (Saputo et al., 2018). *In vitro* studies have revealed that VitD3 application can inhibit virulence factor expression in *Porphyromonas gingivalis* and halt bacterial growth (Grenier et al., 2016). Clinically, individuals with osteoporosis have a higher *H. pylori* infection rate, and those receiving early preventive treatment with VitD3 replenishment have a lower rate of *H. pylori* infection (Chinda et al., 2019; Wang et al., 2019). In addition, the long-term use of 1 α-hydroxyvitamin D_3_ (an analog of VitD3) has been shown to reduce the risk of *H. pylori* infection (Kawaura et al., 2006). 25(OH)D_3_ levels were also shown to be significantly higher in a clinical cohort in which *H. pylori* eradication was successful than in nonresponders (El Shahawy et al., 2018). However, the anti-*H. pylori* effects of VitD3 were not confirmed in a study of Japanese patients in whom a negative correlation was observed between VitD3 serum levels and anti-*H. pylori* effects (Ikezaki et al., 2017). Thus, mechanistic studies are urgently needed to confirm the anti-*H. pylori* effects of VitD3.

VitD3 stimulates innate immune antibacterial activity in a variety of cell types by increasing the production of antimicrobial factors (Hewison, 2011). Generally, VitD3 acts by binding to the vitamin D receptor (VDR) to form a VitD3/VDR complex (Laverny et al., 2009), which further binds to VitD response elements (VDREs), specific DNA sequences upstream of the promoter region of the target genes, to initiate or repress transcription (Laverny et al., 2009). Cathelicidin antimicrobial peptides (CAMPs, also known as LL37, CAP18, or FALL39) are multifunctional antimicrobial peptides that are present in almost all types of vertebrates. CAMPs are primarily expressed by macrophages, dendritic cells, Paneth cells, and epithelial cells of the gastrointestinal tract, respiratory tract, and skin (Vandamme et al., 2012; Xhindoli et al., 2015). As broad-spectrum antimicrobial factors, CAMPs are not only strongly resistant to all types of pathogenic organisms but also active against many antibiotic-resistant clinical bacteria (Gombart et al., 2005). The synthesis and secretion of CAMPs by host cells are significantly enhanced when these cells are exposed to environmental microorganisms and bacteria (Lapis, 2008). As small molecule cationic peptides, CAMPs not only destroy bacteria by directly binding to their cell walls but also inhibit biofilm formation by a variety of bacteria, including *P. aeruginosa, Francisella novisida*, uropathogenic *E. coli*, *S. aureus*, and *Aggregatibacter actinomycetemcomitan* (Golpour et al., 2019). The results of several studies have demonstrated that the anti-infection role of VitD3 against *M. tuberculosis* is closely associated with VitD3/VDR-signal mediated antimicrobial responses and the production of CAMP (Liu et al., 2007). The plasma VitD3 levels were shown to be correlated with local CAMP expression in patients with granulomatous lesions in *M. tuberculosis*-infected lymph nodes (Ashenafi et al., 2018). Furthermore, using the human gastric epithelial cell line GES-1, the VitD3-VDR-CAMP axis has been suggested to be involved in inhibiting *H. pylori* infection (L. Guo et al., 2014), although whether this holds true *in vivo* requires further elucidation. Based on these reports, it has been speculated that directly inducing CAMP production in gastric mucosa is involved in the anti-inflammatory and anti-infection roles of VitD3, which further removes the microorganisms or bacteria from the mucosa and consequently reduces epithelial damage (Krutzik et al., 2008). The use of VitD3, as an effective anti-infection factor, may be a new approach for the development of therapeutics against *H. pylori* infection.

In the present study, we investigated the prophylactic and therapeutic effects of VitD3 against *H. pylori* infection *in vivo* based on a mouse *H. pylori* infection model. Furthermore, a specific contribution of the VitD3/VDR/CAMP pathway in this process was elucidated using a mouse primary gastric epithelial cell *H. pylori* infection model. Our results provided mechanistic evidence in support of the clinical application of VitD3 in the treatment of *H. pylori* infection, especially for patients harboring antibiotic drug-resistant *H. pylori*.

## Materials and Methods

### Bacterial Strains, Cell Culture, and Mice

*H. pylori* Sydney strain (SS)1 (VacA^+^ and CagA^+^) was cultured on Brucella agar medium containing 7% fetal bovine serum, 10 mg/L vancomycin, and 5 mg/L amphotericin at 37°C under a microaerobic humidified atmosphere (5% O_2_, 10% CO_2_, and 85% N_2_) and was identified by Gram staining and PCR. Bacteria were harvested, resuspended in phosphate-buffered saline (PBS, pH 7.4), and then immediately used.

Mouse primary gastric epithelial cells (GECs) were purchased from iCell (Shanghai, China). The cells were grown in epithelial cell culture medium (iCell, Shanghai, China) supplemented with 2% fetal calf serum (iCell, Shanghai, China), 2% epithelial growth factor (iCell, Shanghai, China), penicillin (100 U/mL), and streptomycin (100 μg/mL) at 37°C with 5% CO_2_.

Eight-week-old C57BL/6 mice and VDR knockdown (VDR^-KD^, VDR low expression) mice (C57BL/6 background) were purchased from the Vital River Laboratory Animal Technology Co. Ltd. (Charles River China, Beijing, China) and Biocytogen Co. Ltd. (Beijing, China), respectively. The mice were housed in a specific-pathogen-free environment with free access to food and sterile water. Animal experiments were approved by the Animal Care and Use Committee of Capital Medical University.

### Establishment of Mouse Models of *H. pylori* Infection and VitD3 Treatment

Mouse models of *H. pylori* infection were established according to the method previously reported by Lee (Lee et al., 1997). C57BL/6 or VDR^-KD^ mice were intragastrically inoculated with 0.5 mL of an *H. pylori* suspension (10^9^ CFU/mL, each time) every other day for a total of five times. Before each inoculation, mice were fasted for food and water for 12 and 6 h, respectively. Mice were sacrificed at 8 weeks after *H. pylori* infection. Control mice were inoculated with an equal volume of sterile PBS.

To investigate the inhibitory effect of active vitamin D3 [VitD3, 1,25(OH)_2_D_3_, Sigma-Aldrich (St. Louis, MO, USA)] on *H. pylori* infection, male and female VDR^-KD^ and wild-type (WT) mice (aged 8 weeks) were randomly divided into five groups with five mice in each group. The control group mice were intragastrically administered 0.5 mL of PBS (each mouse) for every other day for a total of five times. Mice in the *H. pylori*-infection group were infected with *H. pylori* suspension as described above, followed by the intragastric administration of 0.5 mL of vegetable oil (each mouse). In the *H. pylori* infection plus VitD3 treatment groups, the infected mice were administered VitD3 at 0.1, 0.4, or 1.6 μg/kg every day for 14 days 8 weeks after infection. During the treatment period, mice were monitored daily. All mice in each group were killed at 10 weeks after infection, and blood and gastric samples were collected for examination.

### Histological Examination by Hematoxylin and Eosin Staining and Transmission Electron Microscopy

At 10 weeks after *H. pylori* infection, a portion of the gastric pyloric tissue was fixed in 4% paraformaldehyde for 24 h, immersed in serial alcohol dehydration solutions and then embedded in paraffin. Subsequently, the tissue sections (4 μm thick) were cut and stained with hematoxylin and eosin (HE) and assessed by light microscopy (Nikon, Eclipse Ni, Japan).

For electron microscopy, the samples were cut into 3–5 mm pieces, placed in 2% paraformaldehyde and 2.5% glutaraldehyde for 2 h at 4°C, and then rinsed three times for 10 minutes each in 0.1 M PBS. Then, ultrathin sections (60 nm) were cut and stained with 1% uranyl acetate and lead citrate before being observed under an HT-7700 transmission electron microscope (HITACHI, Tokyo, Japan).

### Immunofluorescence Staining (IF Staining)

The gastric sections (4 μm thick) were deparaffinized and followed by antigen retrieval with citrate. Nonspecific binding was blocked with 5% bovine serum albumin (Sigma) for 1 h at 25°C. After being incubated with rabbit anti-*H. pylori* antibodies (1:100, ABCAM, Cambridge, MA, USA) overnight at 4°C, the sections were washed and incubated with fluorescein-labeled secondary antibody corresponding to the primary antibody (Alexa Fluor 594-conjugated goat anti-rabbit IgG or Alexa Fluor 488-conjugated anti-mouse IgG) (Life Technologies, Waltham, MA, USA) at 25°C for 1 h in the dark. Then, the cell nuclei were stained with DAPI, and the sections were observed using a fluorescence microscope (IX83, FLUOVIEW FV1200, Olympus).

### Immunohistochemistry (IHC)

After being deparaffinized and washed with PBS, the sections were treated with 3% H_2_O_2_ followed by citrate for antigen retrieval. Nonspecific binding was blocked by incubating the sections in 5% goat serum and 1% bovine serum albumin in PBS for 1 h at 25°C, after which they were incubated with mouse anti-VDR or anti-CAMP antibodies (Santa Cruz Biotechnology Inc., CA, USA) at 4°C overnight. Subsequently, after being washed with PBS, the sections were incubated with HRP-conjugated goat anti-mouse IgG and visualized with 0.01% H_2_O_2_/0.05% 3,3-diaminobenzidine reagents.

### RNA Extraction and Quantitative Real-Time PCR (RT-qPCR) Assay

Total RNA was extracted from mouse GECs or gastric mucosal tissues using the TRIzol reagent (Invitrogen, Waltham, MA, USA) according to the manufacturer’s instructions. Reverse transcription reactions were performed using PrimeScript™ RT Master Mix (TAKARA, China, Dalian), with 20-μL reactions incubated in a Veriti 96-well Thermal Cycler (Applied Biosystem™, Waltham, MA, USA) for 15 min at 37°C and 5 s at 85°C. Quantitative real-time PCR was performed in an ABI 7500 real-time PCR system (Applied Biosystem™, Waltham, MA, USA) using SYBR Green mix (Invitrogen) with primers listed in **Table 1**. The amplification parameters were as follows: 95°C for 2 min; followed by 40 cycles of 95°C for 15 s, 56°C for 20 s, and 72°C for 30 s; with a final incubation at 72°C for 2 min. All PCRs were performed in triplicate. Gene expression levels were normalized to that of GAPDH, and the 2^−ΔΔCt^ method was used for statistical analysis.

**Table 1:** Primer for Real-time PCR.

Name of Primer	Sequence
VDR	5’ - ACCCTGGTGACTTTGACCG -3’
	5’ - GGCAATCTCCATTGAAGGGG -3’
CAMP	5’ - GCTGTGGCGGTCACTATCAC - 3’
	5’ – TGTCTAGGGACTGCTGGTTGA - 3’
GAPDH	5’ - AGGTCGGTGTGAACGGATTTG -3’
	5’ - TGTAGACCATGTAGTTGAGGTCA - 3’

### Western Blot Analysis (WB)

Total protein was extracted from mouse GECs or gastric mucosal tissues that were pretreated with RIPA lysis buffer (50 mM Tris/HCl, 150 mM NaCl, 1% NP-40, 0.5% sodium deoxycholate, and 0.1% SDS) and disrupted with gentle sonication (on 3 s/off 3 s for 10 cycles), after which the samples were centrifuged at 12,000 rpm for 15 minutes to pellet the cell debris. A bicinchoninic acid (BCA) protein assay kit (Thermo Fisher, Waltham, MA, USA) was used to measure protein concentrations. Equal amounts of protein were separated by 12% SDS/PAGE and then transferred to polyvinylidene fluoride membranes. Then, the membranes were blocked for 2 h with 5% (w/v) nonfat milk in TBST, after which they were incubated with the primary antibodies listed in **Table 2** overnight at 4°C. After being washed with TBST, the membranes were incubated with the appropriate HRP-conjugated secondary antibodies at 25°C for 1 h, visualized using by enhanced chemiluminescence and viewed with a Bio-Rad system (Hercules, CA, USA).

**Table 2:** Information of primary antibodies used in WB and IF/IHC.

Primary antibody	Company	Cataloguenumber	Dilution factor		
			Western blot	Immunofluorescence	Immunohistochemistry
GAPDH	Proteintech	60004-1-1g	1:5000		
VDR	Santa Cruz.	sc-13133	1:1000	1:100	1:200
CAMP	Santa Cruz.	sc-166055	1:1000	1:100	1:200
CagA	Santa Cruz.	sc-28368	1:1000		
*H.pylori*	ABCAM	ab20459		1:500	

### ELISA

Mouse IL-6/IL-8 ELISA kits were purchased from Andygene Biotechnology (Beijing, China). Mouse blood was collected from the periocular venous plexus and centrifuged to obtain serum. The assay was performed according to the manufacturer’s instructions. In brief, the standard and serum samples were added to a microplate well coated with antibodies to IL-6 or IL-8 and were sequentially incubated with different reagents. Then, the absorbance values at 450 nm were determined and compared with those of known standards.

### Mouse GECs Infected With *H. pylori* and Treatment With VitD3

To determine the optimal concentration of VitD3 for subsequent experiments, mouse GECs were treated with VitD3 at various concentrations (20-100 nM) for 48 h and then collected to assess VDR and CAMP expression by Western blot (WB) analysis. All VitD3 treatment experiments were performed in triplicate.

The cultured mouse GECs were treated with 10^8^ CFU/mL of *H. pylori* at MOI=100. At 2 h after *H. pylori* infection, VitD3 (60 nM) was added to the cultured cells and then incubated for 6 h. Subsequently, the cell samples were harvested and processed for examination of CagA, VDR, and CAMP protein expression by Western blot analysis.

### Luciferase Reporter Assay

The mouse gene encoding VDR was amplified and cloned into the vector pcDNA3.1. The pGL3 plasmid carrying either the CAMP promoter region [from -2000 base pairs to the transcription start site (TSS)] or the mutant VDR binding site as well as luciferase reporter gene were constructed by YouBio (Changsha, China).

For transfection, HEK-293T cells seeded in 24-well plates with or without VitD3 medium were cotransfected with 2 μg of pcDNA3.1-VDR, 1 μg of the luciferase reporter plasmid, and 200 ng of the pRL-TK plasmid using Lipofectamine 3000 according to the manufacturer’s instructions. After culturing at 37°C for 48 h, the luciferase activity was assessed according to the standard protocol of the Promega Dual Luciferase Assay System (Thermo Fisher). Luciferase activity values were normalized to the corresponding Renilla luciferase activity values.

### Statistical Analysis

All data were analyzed and visualized using GraphPad Prism 8. The results are presented as the means ± standard error of the mean (SEM). Student’s t-test was used to compare quantitative data between two groups. Two-way ANOVA and multiple t-test were used to compare grouped quantitative data. *P*-values < 0.05 were considered statistically significant.

## Results

### Treatment With VitD3 (1,25(OH)_2_D_3_) Inhibits *H. pylori* Infection in Mice

In our culture system, *H. pylori* SS1 with white needle tip colonies were observed after 72 h. Typical spiral-shaped morphology and 550-bp amplified fragments were observed by Gram staining and PCR analysis with specific primers (**Figure S1**). After being harvested, the bacteria were immediately used for subsequent assays.

**Figure 1:**
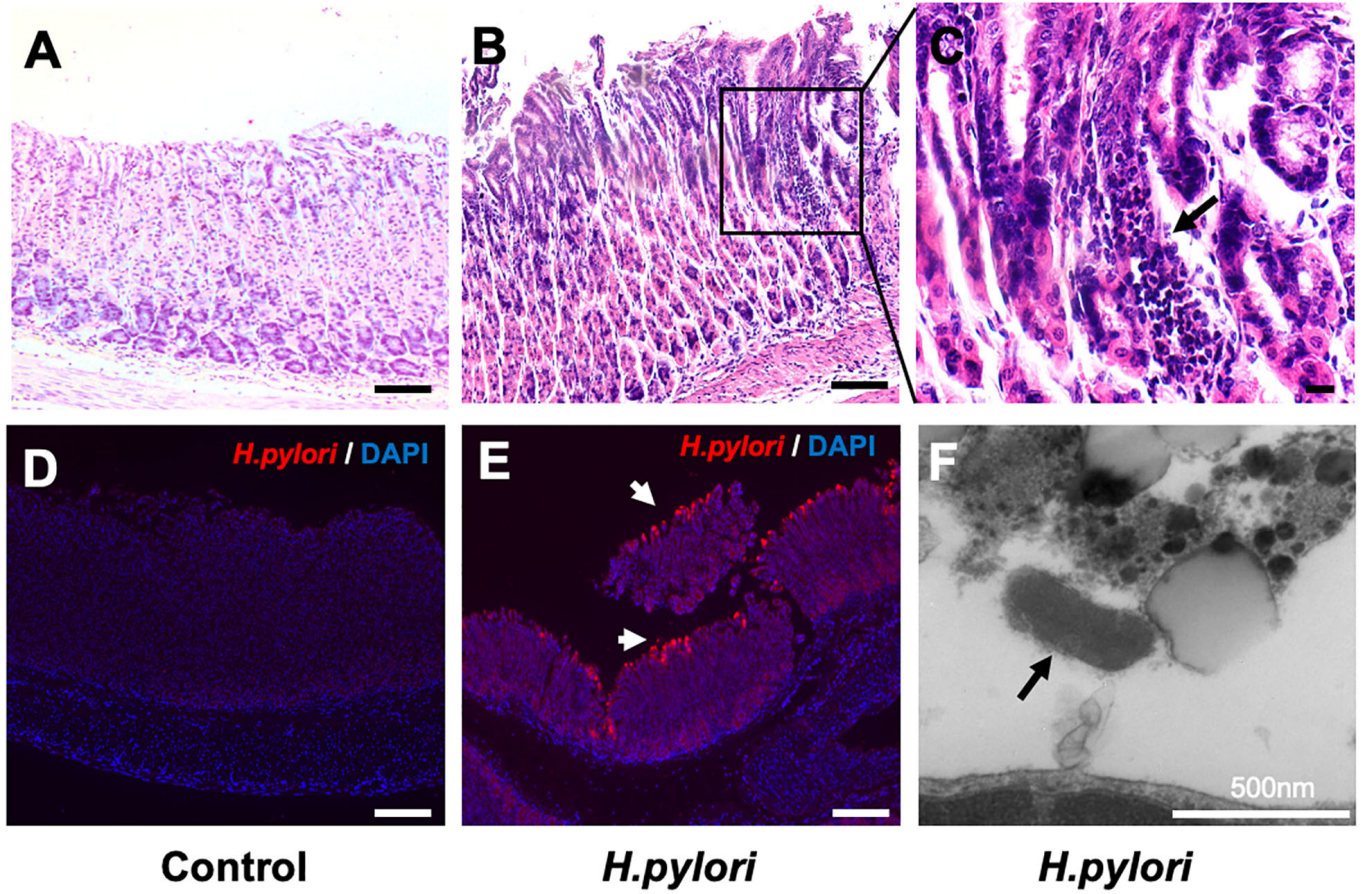
Establishment of a mouse model of *Helicobacter pylori* infection. **(A–C)** Hematoxylin and eosin staining results showed gastric mucosal epithelial inflammation (black arrow) at 8 weeks after *H. pylori* gavage in mice. Scale bar=100 μm. **(D, E)** Immunofluorescence staining results indicated that *H. pylori* (red, white arrow) colonized the surface of gastric mucosa in infected mice. DAPI was used to stain nuclei. Scale bar=100 μm. **(F)** The colonization of rod-shaped *H. pylori* (black arrow) on the surface of the gastric mucosa was observed by transmission electron microscopy. Scale bar=500 nm.

To assess whether VitD3 has a role in anti-*H. pylori* infection *in vivo*, a mouse model was developed. At 8 weeks after *H. pylori* infection, the stomach samples were collected to assess *H. pylori* infection and gastric inflammation. Compared to that observed in the control mice (**Figure 1A**), large amounts of inflammatory cell infiltration and congestion were observed in the gastric mucosa of the infected mice (**Figures 1B, C**), indicating that chronic inflammation had occurred. Furthermore, IF staining results showed *H. pylori* colonization on the surface of gastric mucosa in the infected mice (**Figure 1E**), whereas no specific fluorescence was observed in the control mice (**Figure 1D**). TEM results further revealed the colonization of rod-shaped *H. pylori* cells on the surface of gastric mucosa (**Figure 1F**). Collectively, these results demonstrate that the *H. pylori* infection mouse model was established.

**Figure 2:**
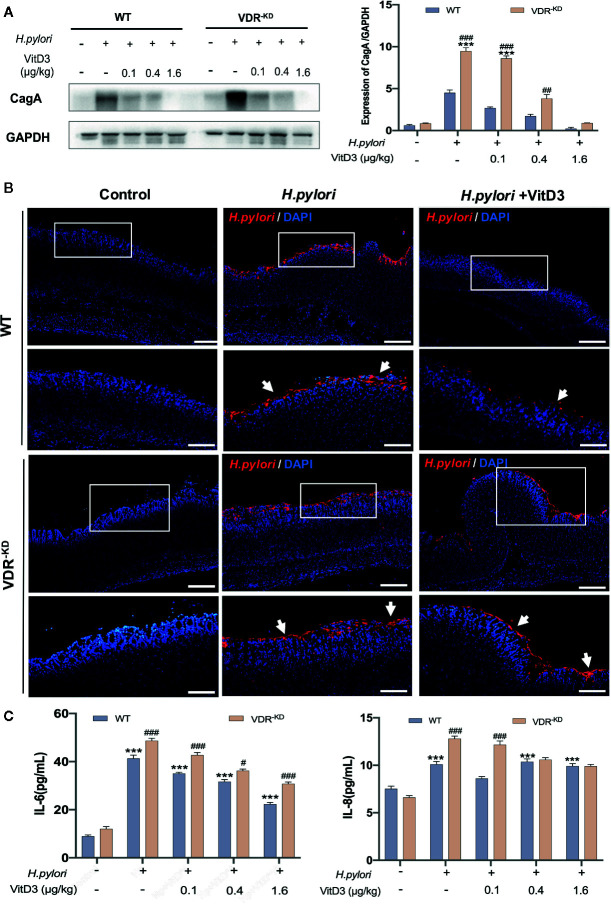
VitD3 inhibits *H. pylori* infection in mice. Different doses of VitD3 were administered at 8 weeks after *H. pylori* infection for 14 days. **(A)** Western blot results showing CagA expression in WT and VDR^-KD^ mice in all groups, including the control, *H. pylori* infection alone and *H. pylori* infection plus VitD3 treatment groups. Compared with that observed in the *H. pylori*-infected group, CagA expression in the VitD3-treated group was significantly reduced in a dose-dependent manner (n=5, *p*<0.05). **(B)** IF staining results showed that *H. pylori* colonization (red, white arrow) was reduced by VitD3 administration compared to that observed in the *(H)* *pylori*-infected groups for both the WT and VDR^-KD^ mice. Scale bar=100 μm. **(C)** The serum levels of IL-6 and IL-8 detected by ELISA were higher in VDR^-KD^ mice than in WT mice; VitD3 treatment inhibits IL-6 and IL-8 production in a dose-dependent manner in WT mice (n=5, ^#^ *p* < 0.05, ^##^ *p* < 0.01, ^###^ *p* < 0.001, ***p < 0.001 *** vs. WT control; ^###^WT vs. VDR^-KD^).

Next, *H. pylori*-infected mice were treated with or without VitD3 for 14 days, and the expression of CagA, a protein toxin of *H. pylori*, in mouse gastric mucosa was measured to evaluate the effect of VitD3 on *H. pylori* infection. As shown in **Figure 2A**, in control WT mice without VitD3 treatment, a large amount of CagA was detected. In contrast, CagA expression in WT mice treated with VitD3 was significantly lower than that observed in the control mice. Furthermore, the decrease in CagA levels was associated with VitD3 treatment in a dose-dependent manner. In particular, for mice treated with 1.6 μg/kg of VitD3, CagA expression was almost undetectable. Consistent with these results, IF staining also showed a reduced specific immune reaction for *H. pylori* after VitD3 treatment (**Figure 2B**, upper row) compared to that observed in the control group. The results indicated that VitD3 has an anti-Hp infection role *in vivo*.

### Anti-*H. Pylori* Infection Activity of Vitd3 Is Associated With Augmented VDR Expression *In Vivo*

VitD3 is known to play various biological effects by binding to its receptor VDR. VDR knockdown (VDR^-KD^) mice were thus constructed in a C57/B6 background and routinely genetically verified by PCR with specific primers (**Figure S2**). Subsequently, these mice were used to further study mechanism underlying the anti-*H. pylori* infection function of VitD3. When *H. pylori*-infected VDR^-KD^ mice were treated with VitD3, similar inhibitory effects on *H. pylori* infection were observed, as revealed by CagA detection and IF staining results (**Figures 2A, B**, bottom row), which also showed a dose-dependent effect of VitD3. Under *H. pylori* infection conditions, however, regardless of treatment with or without VitD3, the CagA content in VDR^-KD^ mice was significantly higher than that observed in WT mice, indicating that VDR knockdown increased the susceptibility of mice to *H. pylori* infection (**Figures 2A, B**) and that VitD3 plays an anti-*H. pylori* infection role *via* VitD3/VDR signaling.

In addition, increased serum IL-6 and IL-8 levels were observed in both WT and VDR^-KD^ mice after *H. pylori* infection, and VitD3 treatment suppressed the levels of IL-6 and IL-8 (**Figure 2C**), further suggesting that VitD3 could suppress *H. pylori* infection and inhibit inflammation.

**Figure 3:**
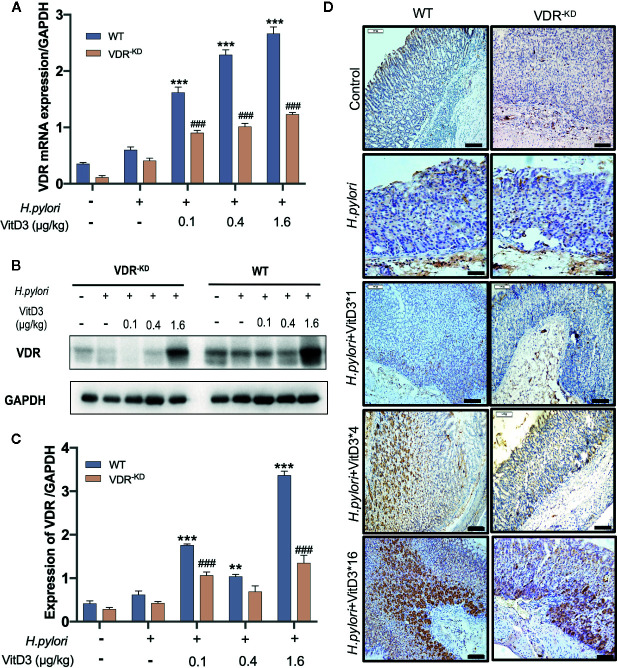
*Anti-H. pylori* infection activity of VitD3 is associated with enhanced VDR expression *in vivo.* Different doses of VitD3 were administered at 8 weeks after *H. pylori* infection for 14 days. **(A, B)** RT-qPCR or Western blot analyses was used to assess VDR expression in WT and VDR^-KD^ mice in all groups, including the control, *H. pylori* infection alone and *H. pylori* infection plus VitD3 treatment groups. VitD3 treatment significantly increased VDR expression in a dose-dependent manner in both mouse models. VDR expression in VDR^-KD^ mice was significantly lower than that observed in WT mice (n=5, *p*<0.05). **(C)** The histogram shows the gray values represented for the results presented in **(B)**. **(D)** Immunohistochemical staining results showing VDR expression in both the WT and VDR^-KD^ mouse models in all groups. VDR expression increased after *H. pylori* infection, and VitD3 treatment promoted this expression in a dose-dependent manner. **p < 0.01, ***p < 0.001, ^###^ *p* < 0.001. All values are presented as the means ± SEM from 5 independent experiments unless otherwise stated. Scale bar= 50 μm.

To further confirm that VitD3 inhibits *H. pylori* infection *in vivo* by interacting with VDR, gastric samples of control mice, and *H. pylori*-infected mice treated with or without VitD3 were examined for VDR expression. As expected, a specific level of VDR expression in gastric mucosa was detected in control mice, but VitD3 treatment alone (0.4 μg/kg per mouse, for 14 days) could significantly increase VDR expression (**Figure S3**). IF staining results showed that VDR was primarily distributed in the lower and middle parts of the gastric fundic gland (**Figure S4**, upper row). *H. pylori* infection had no obvious effect on VDR expression (**Figures 3A–C**). After VitD3 treatment for 14 days, however, significantly elevated VDR protein expression and mRNA levels in gastric mucosa were observed in both WT and VDR^-KD^ mice (**Figures 3A–C**), accompanied by reduced CagA levels and *H. pylori* colonization (**Figures 2A, B**). Furthermore, IHC results showed that the increased staining intensity of VDR was closely associated with the amount of VitD3 used (**Figure 3D**), with the highest VDR expression observed when 1.6 μg/kg of VitD3 was used. However, in VDR^-KD^ mice, the levels of VDR mRNA and protein were significantly lower than those observed in WT mice after treatment with VitD3 (n=5, *p*<0.05) (**Figures 3A–C**). Taken together, our results indicated that VitD3 can enhance VDR expression *in vivo*.

### VitD3 Treatment Enhances CAMP Expression *In Vivo*

**Figure 4:**
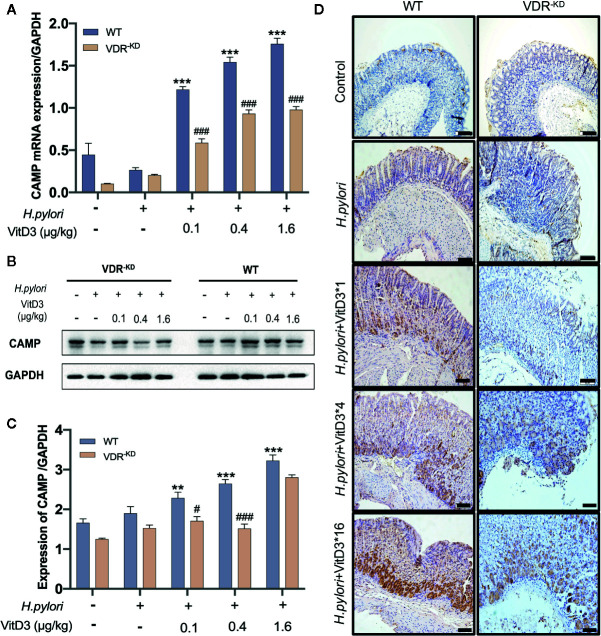
CAMP is a target gene activated by VitD/VDR signaling. Different doses of VitD3 were administered at 8 weeks after *H. pylori* infection for 14 days. **(A)** and **(B)** RT-qPCR or Western blot analyses were performed to assess CAMP expression in WT and VDR^-KD^ mice in all groups, including the control, *H. pylori* infection alone and *H. pylori* infection plus VitD3 treatment groups. VitD3 treatment significantly increased CAMP expression in a dose-dependent manner in both mouse models. CAMP expression in VDR^-KD^ mice was significantly lower than that observed in WT mice (n=5, *p*<0.05). **(C)** The histogram shows the gray values for the results presented in **(B)**. **(D)** Immunohistochemical staining results showed CAMP expression in both the WT and VDR^-KD^ mouse models in all groups mentioned above. CAMP expression increased after *H. pylori* infection, and VitD3 treatment promoted this expression in a dose-dependent manner. ^#^ *p* < 0.05, ^###^ *p* < 0.001, **p < 0.01, ***p < 0.001. All values are presented as the means ± SEM from 5 independent experiments unless otherwise stated. Scale bar= 50 μm.

To elucidate the mechanisms underlying the anti-*H. pylori* infection activity of VitD3 *in vivo*, we searched for target genes of the VitD3/VDR complex and elected to focus on CAMP due to its strong antibacterial effect. CAMP expression in gastric mucosa was examined and could be detected in the healthy mice (**Figures 4A–C**, and **S4**, bottom row). *H. pylori*-infection, however, slightly enhanced CAMP expression at both the mRNA and protein levels in both WT and VDR^-KD^ mice (**Figures 4A–C**). In line with the VDR expression data described above, VitD3 treatment significantly increased CAMP expression in a dose-dependent manner in WT mice, as revealed by WB and RT-qPCR assay results (**Figures 4A–C**) and was confirmed by IHC staining with an antibody to CAMP (**Figure 4D**). In addition, VitD3 treatment also increased the expression of CAMP in VDR^-KD^ mice with a similar pattern, but the magnitude was lower than that observed in WT mice (n=5, *p*<0.05). Our results indicated that CAMP is a target gene of VitD3/VDR signaling and plays a role in anti-*H. pylori* infection.

### CAMP Is a Direct Target Gene of the Vitd3/VDR Signaling Pathway

**Figure 5:**
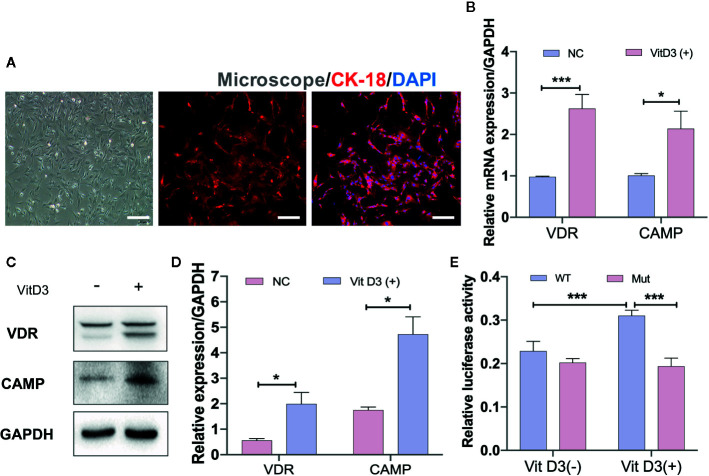
VitD3 enhances VDR and CAMP expression *in vitro*. **(A)** Primary mouse gastric epithelial cells were cultured and identified using an antibody to keratin 18 (red) by IF staining. DAPI was used to stain cell nuclei. Scale bar=100 μm. RT-qPCR **(B)** and Western blot **(C)** analyses were used to assess VDR and CAMP expression after treatment with 60 nM VitD3 for 48 h. **(D)** The histogram shows the gray values for the results presented in **(C)**. **(E)** Luciferase assays were performed using HEK-293T cells, and the results indicated that luciferase activity was significantly increased after VitD3 treatment in cells harboring WT but not mutant CAMP promoter reporter plasmids. *p<0.05, ***p<0.001. All values are presented as the means ± SEM from 3 independent experiments unless otherwise stated.

To further confirm that CAMP is directly associated with the VitD3/VDR signaling pathway, mouse primary gastric epithelial cells (GECs) were cultured to differentiate into spindle shape and were demonstrated by IF staining to be positive for antibodies against keratin 18 (CK-18), a gastric epithelial-specific marker (**Figure 5A**). When mouse GECs were treated with exogenous VitD3 at 20–100 nM for 48 h, the expression of both VDR and CAMP proteins were significantly increased in a VitD3 dose-dependent manner (**Figure S5**). Because the increased VDR and CAMP protein levels plateaued at 60–100 nM of VitD3, 60 nM was used in subsequent experiments. As expected, the mouse GECs treated with VitD3 (60 nM) exhibited significantly enhanced gene transcription and protein expression of both VDR and CAMP (**Figures 5B–D**), suggesting that VitD3 induced the production of VDR and CAMP *in vitro*.

Next, luciferase reporter gene analysis was performed to examine whether VDR directly binds to the CAMP promoter region using HEK-293T cells, which were first transfected with the mouse VDR gene (**Figure S6A, B**). When cotransfected with the WT reporter plasmid (Materials and Methods), luciferase enzyme activity was significantly increased after treatment with VitD3, whereas cotransfection with the mutant reporter plasmid that abolished binding sites between them had no influence on luciferase activity after VitD3 treatment (**Figure 5E**). These results suggested that VDR can directly interact with the -1649 to -1664 bp upstream region of the transcriptional start site of the CAMP promoter region (**Figure S6C**).

**Figure 6:**
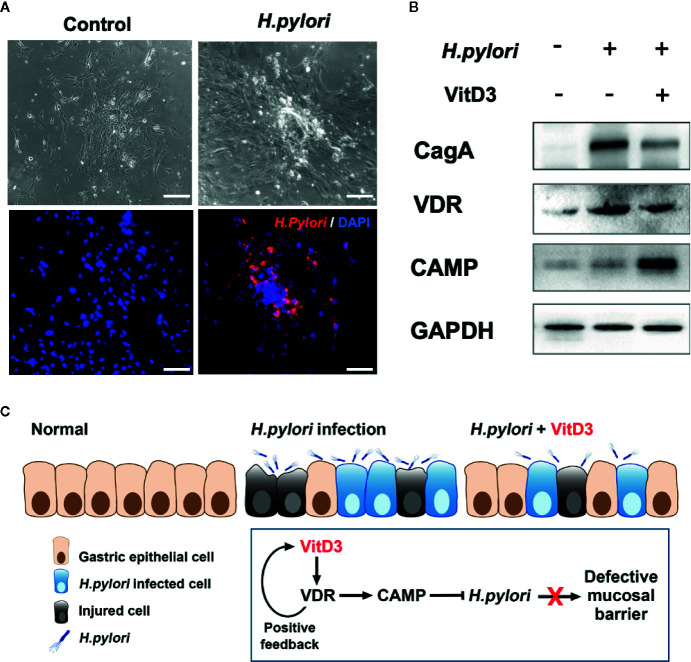
*H. pylori* infection promotes VDR and CAMP expression. **(A)** *H. pylori* infection model mouse gastric epithelial cells were infected with *H. pylori* (MOI=100). Morphological changes and successful infection were revealed by microscopic observations and IF staining using a specific anti-*H. pylori* antibody (red). DAPI was used to stain cell nuclei. Scale bar=100 μm. **(B)** Western blot results showed the expression of VDR and CAMP in *H. pylori*-infected mouse gastric epithelial cells treated with or without VitD3 (60 nM). VDR and CAMP expression was significantly enhanced by the addition of VitD3, which was accompanied by decreased in CagA expression. **(C)** A graphic representation of the possible pathogenesis mechanism associated with *H. pylori* infection and VitD3 treatment in the mouse stomach.

To further elucidate the anti-*H. pylori* infection activity of VitD3, mouse GECs were exposed to *H. pylori* at an MOI of 100 for 6 h. A large amount of *H. pylori* adhered to the surface of the cultured cells, which was accompanied by cell morphological changes such as cell denaturation and death, indicating cell damage (**Figure 6A**). Notably, VDR and CAMP expression was significantly increased after *H. pylori* infection and was more pronounced by treatment with VitD3, where decreased CagA levels were also observed (**Figure 6B**). In line with the *in vivo* results, these data further suggested that *H. pylori* infection could induce the expression of both VDR and CAMP and that their expression was enhanced treated with VitD3. In other words, VitD3 exerts an antibacterial effect on *H. pylori via* the VitD3/VDR/CAMP pathway.

## Discussion

*H. pylori* infection is an important clinical problem for which the current antibiotic treatment is not entirely satisfactory, as antimicrobial resistance of the commonly used agent hampers effective antimicrobial treatment. Recently, the WHO published a list of the 12 most crucial multidrug resistant bacterial groups, which includes clarithromycin-resistant *H. pylori* (Tacconelli, 2017). As an alternative or adjunct therapy, antibiotic-independent candidates with antimicrobial properties may be needed to combat *H. pylori* infection. VitD3 is an important biologically active substance without obvious side effects. In addition to modulating the metabolism of calcium and phosphorus, its antimicrobial and immunomodulatory functions have been demonstrated (Golpour et al., 2019). For example, VitD3 has been reported to be an effective agent to treat tuberculosis by inhibiting mycobacterial lipoarabinomannan and reducing inflammation in the lungs of patients (Liu et al., 2007; Schauber et al., 2007), with similar effects having been shown toward other bacteria, such as *E*. *coli*. and *P*. *aeruginosa*. Regarding the effects of VitD3 on *H. pylori* infection, however there are some conflicting results from *in vivo* studies, and the underlying mechanism is not fully understood.

In the present study, we established mouse *H. pylori* infection models in both WT and VDR^-KD^ mice. These mice were used to demonstrate that VitD3 inhibits *H. pylori* infection by enhancing the expression of VDR and CAMP and that VDR^-KD^ mice with lower VDR expression are more susceptible to *H. pylori* infection. Using cultured mouse primary gastric epithelial cells, we further demonstrated that the associated mechanism involves binding of the VitD3/VDR complex to the CAMP promoter region to increase its expression.

Our results are supported by those of a previous study in which CAMP knockout mice were shown to be significantly more susceptible to *H. pylori* infection and exhibited more severe gastric mucosal inflammation compared to that observed in WT mice. Furthermore, supplementation of exogenous CAMP was shown to alleviate inflammation, reduce *H. pylori* colonization in the gastric mucosa and decrease the production of inflammatory cytokines in mice (Zhang et al., 2013), suggesting that CAMP plays an important role in inhibiting *H. pylori* infection in mouse gastric mucosa. Consistently, treatment of *H. pylori*-infected human gastric mucosal epithelial cell line GES-1 with VitD3 could upregulate VDR expression and CAMP secretion and suppress *H. pylori* replication (L. Guo et al., 2014). Taken together, these results and those of the present study suggest that VitD3 inhibits *H. pylori* infection by enhancing CAMP secretion.

The link between CAMPs and VitD3 is not entirely clear and may be associated with a number of mechanisms. For instance, VitD3 can synergize with 4-phenylbutyrate, a substance that can induce the expression of CAMPs, and VitD3 itself can also upregulate the expression of the CAMPs (Steinmann et al., 2009). Previously, Gombart *et al*. demonstrated that there is a consensus VitD response element (VDRE) in the CAMP promoter that can be bound by VDR, which strongly upregulates CAMP expression in myeloid cells treated with VitD3 (Wang et al., 2004; Gombart et al., 2005; Guo et al., 2013). Wang *et al*. showed, however, that VDREs appear to not be conserved between humans and mice. They speculated that VDREs located in the short interspersed nuclear element (SINE), which is evolutionarily conserved in mammals, were absent in rodents such as mice, rats, and canines (T. T. Wang et al., 2005). Therefore, the mechanism underlying the anti-*H. pylori* infection activity of VitD3 *in vivo* remains unresolved.

In the present study, we observed that VitD3 treatment significantly increased the expression of VDR and CAMP in both WT and VDR^-KD^ mice, indicating an interaction between VitD3/VDR and CAMP in mice. Since previous studies primarily focused on natural DR-3 VDREs not further than -1000 bp upstream from the transcriptional start site (Bouvard et al., 2009), and because there may be some upstream of this region that were ignored, we expanded the search region for VitD3/VDR complex-binding sites to include further upstream of the CAMP promoter in the present study. Using the JASPAR database, we identified a possible binding site in the region -2000 bp upstream from the transcriptional start site at -1649 bp. Furthermore, through luciferase reporter gene analysis, we demonstrated that the VitD3/VDR complex can directly bind to this site in the CAMP promoter region, resulting in a significant increase in luciferase activity in cells treated with VitD3. Our results suggested that VitD3 can reduce *H. pylori* infection in gastric mucosa in mice *via* direct upregulation of CAMP expression (**Figure 6C**), promoting a better understanding of the mechanism underlying VitD3 anti-*H. pylori* activity and suggesting novel approaches for expanding the current clinical treatment of drug-resistant *H. pylori* infection.

There are some limitations to the present study. First, we only demonstrated the role of CAMP in anti-*H. pylori* activity induced by VitD3 and identified a new binding site of VitD3/VDR in the CAMP promoter region. The anti-*H. pylori* mechanism of VitD3 action is known to be complex. In addition to CAMP, 6 α-defensins and 4 β-defensins with anti-microbial properties are also induced by the VitD3 intracrine system and can synergistically function with CAMP to disrupt microbial membranes and stimulate autophagy (Schwalfenberg, 2011). The results of recent studies have suggested that VitD3 can restore lysosomal degradation function by activating the protein disulfide isomerase family A member 3 (PDIA3) receptor, which leads to enhanced Ca^2+^ release from lysosomes and normalization of lysosomal acidification, eventually eliminating *H. pylori* hiding in autophagosomes (Hu et al., 2019). Therefore, further studies are needed to identify additional possible mechanisms concerning the VitD3-mediated eradication of *H. pylori* antibiotic-resistant strains. Second, although studies have shown that VitD3 treatment can reduce *H. pylori* colonization in the stomachs of infected mice, only a few clinical trials have investigated the correlation between VitD3 and *H. pylori* eradication rates, and the conclusions reached are inconsistent (Ikezaki et al., 2017; El Shahawy et al., 2018). Large-scale clinical trials should therefore be conducted in the future to verify its effectiveness.

In summary, through assays involving the successful establishment of an animal model of *H. pylori* infection and cultured primary mouse GECs, we demonstrated that VitD3 can significantly increase VDR and CAMP expression in the gastric mucosa and reduce *H. pylori* infection in mice. Furthermore, we identified a new binding site of the VitD3/VDR complex at the distal end of the CAMP promoter region (position -1649 bp), which may be an important mechanism for the anti-*H. pylori* infection activity of VitD3. Our results offer a new explanation for the clinical application of VitD3 in *H. pylori* eradication therapy.

## Data Availability Statement

All datasets presented in this study are included in the article/**Supplementary Material**.

## Ethics Statement

The animal study was reviewed and approved by Animal Care and Use Committee of Capital Medical University (Permit Number AEEI-2017-032, 19 March 2017).

## Author Contributions

SZha and PL conceived and designed the study. CL provided H. pylori SS1 for following experiments. SL and GZ designed and performed animal experiments. QG guided other biological experiments. AZ and LL performed all other experiments, statistical analysis, and wrote the paper. LM and SZhu reviewed and edited the manuscript. All authors contributed to the article and approved the submitted version.

## Funding

This work was supported in part by grants from the National Natural Science Foundation of China (81570507), The Digestive Medical Coordinated Development Center of Beijing Municipal Administration of Hospitals (XXZ01, XXZ02) and National Key Research and Development Program of China (2017YFC0113600).

## Conflict of Interest

The authors declare that the research was conducted in the absence of any commercial or financial relationships that could be construed as a potential conflict of interest.

## Supplementary Material

Supplementary Figure 1
**(A)** The cultured *H. pylori* strain SS1 showed typical morphology with white needle tip colonies and identified by Gram staining and PCR with specific primers. **(B)** The *H. pylori* strain was identified by PCR using specific primers.

Supplementary Figure 2 VDR knockdown heterozygous mice (VDR^-KD^) were routinely genetically identified by PCR.

Supplementary Figure 3 Western blot results showed the expression levels of VDR protein in WT mice treated with or without VitD3 (0.4 μg/kg). VDR expression was significantly increased in the VitD3 treatment group.

Supplementary Figure 4 IF staining results showing that VDR (green, upper row) and CAMP (green, bottom row) are primarily distributed in the lower and middle parts of the fundic gland and in the whole layer of gastric mucosa, respectively. Scale bar=50 μm.

Supplementary Figure 5 Western blot results showing that VitD3 treatment enhances the protein levels of VDR and CAMP in dose-dependent manner in mouse gastric epithelial cells.

Supplementary Figure 6 RT-qPCR **(A)** and Western blot **(B)** results revealing successful transfection of the VDR gene into HEK-293T cells. **(C)** The sequence of the luciferase reporter plasmid (pGL3) harboring the CAMP promoter region with the WT sequence or the mutant VDR binding site.

## Abbreviations

H. pylori, Helicobacter pylori; VDR, Vitamin D receptor; CAMP, Cathelicidin antimicrobial peptide; VDR^-KD^, VDR knockdown; GECs, Gastric epithelial cells; VDRE, Vitamin D response element; SINE, Short interspersed nuclear element.
