# Supplementary figures and images for: Vitamin D3 Inhibits Helicobacter pylori Infection by Activating the VitD3/VDR-CAMP Pathway in Mice
Source: Front Cell Infect Microbiol. 2020 Oct 23;10:566730. doi: 10.3389/fcimb.2020.566730 (PMC7646218; doi:10.3389/fcimb.2020.566730)

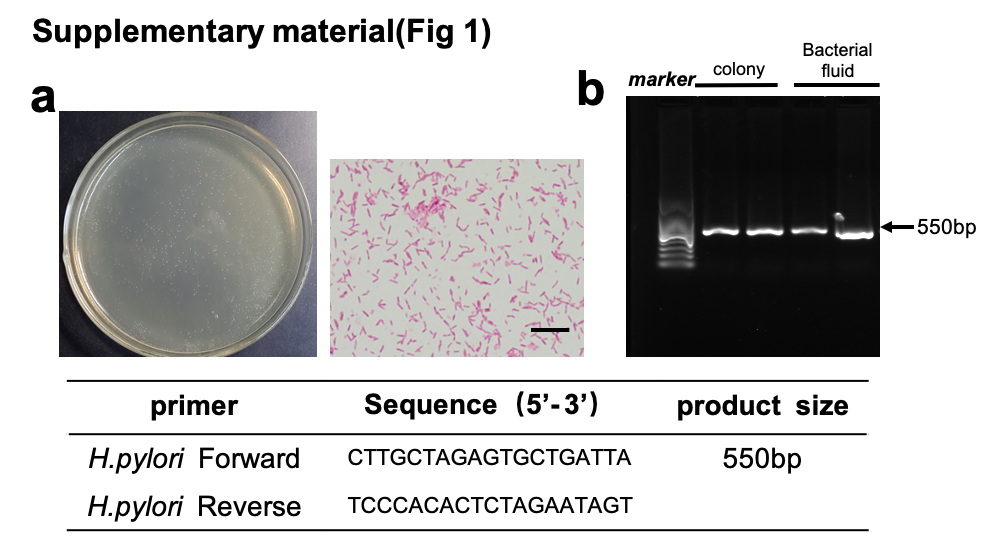

Supplement: Supplementary Figure 1 — (A) The cultured H. pylori strain SS1 showed typical morphology with white needle tip colonies and identified by Gram staining and PCR with specific primers. (B) The H. pylori strain was identified by PCR using specific primers. [file SupplementaryFigure_1.png]

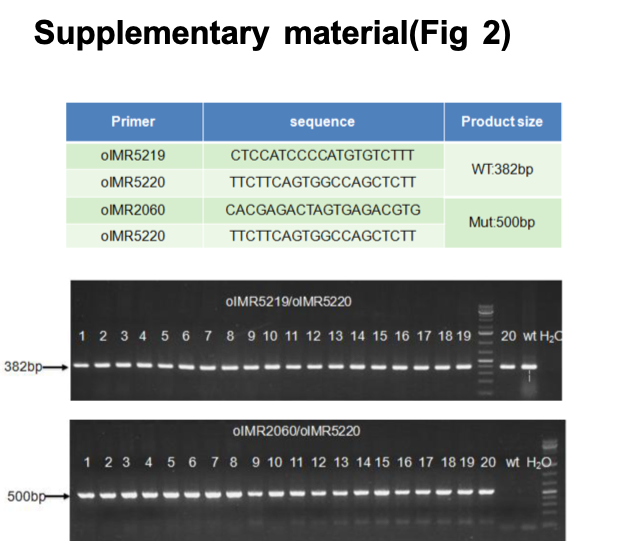

Supplement: Supplementary Figure 2 — VDR knockdown heterozygous mice (VDR-KD) were routinely genetically identified by PCR. [file SupplementaryFigure_2.png]

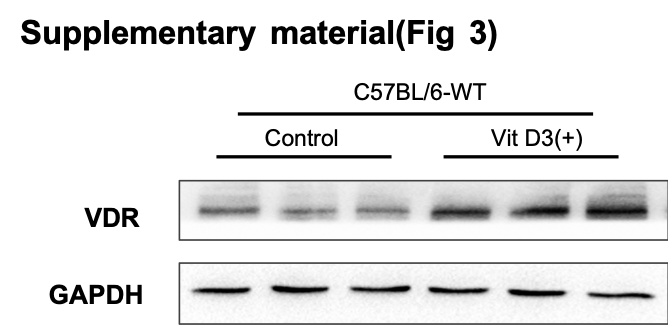

Supplement: Supplementary Figure 3 — Western blot results showed the expression levels of VDR protein in WT mice treated with or without VitD3 (0.4 μg/kg). VDR expression was significantly increased in the VitD3 treatment group. [file SupplementaryFigure_3.png]

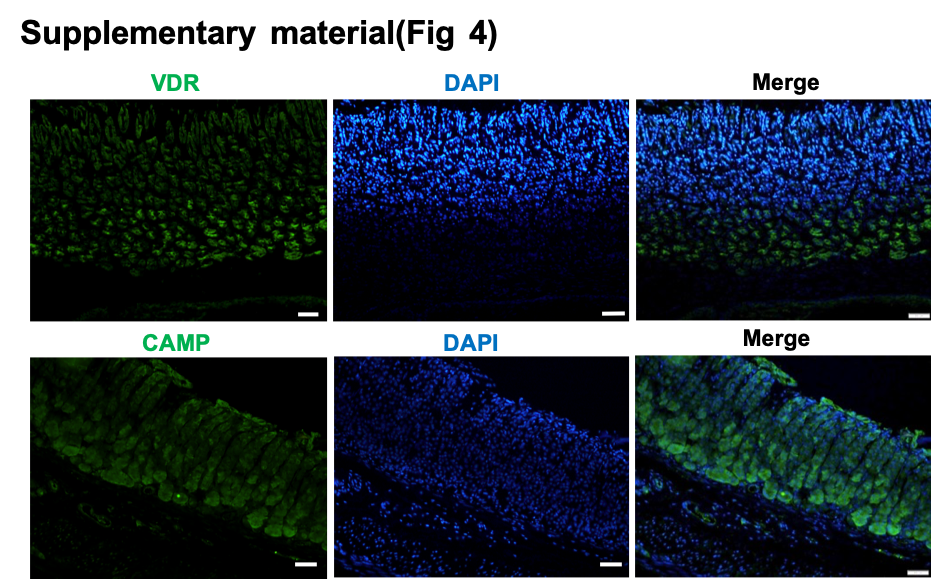

Supplement: Supplementary Figure 4 — IF staining results showing that VDR (green, upper row) and CAMP (green, bottom row) are primarily distributed in the lower and middle parts of the fundic gland and in the whole layer of gastric mucosa, respectively. Scale bar=50 μm. [file SupplementaryFigure_4.png]

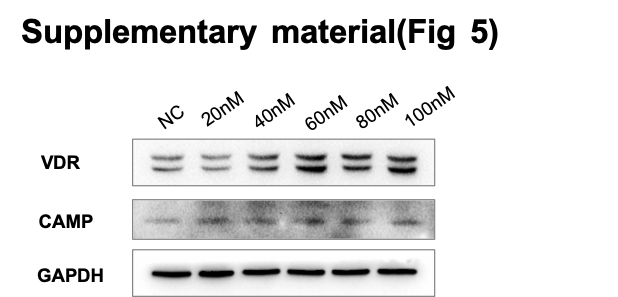

Supplement: Supplementary Figure 5 — Western blot results showing that VitD3 treatment enhances the protein levels of VDR and CAMP in dose-dependent manner in mouse gastric epithelial cells. [file SupplementaryFigure_5.png]

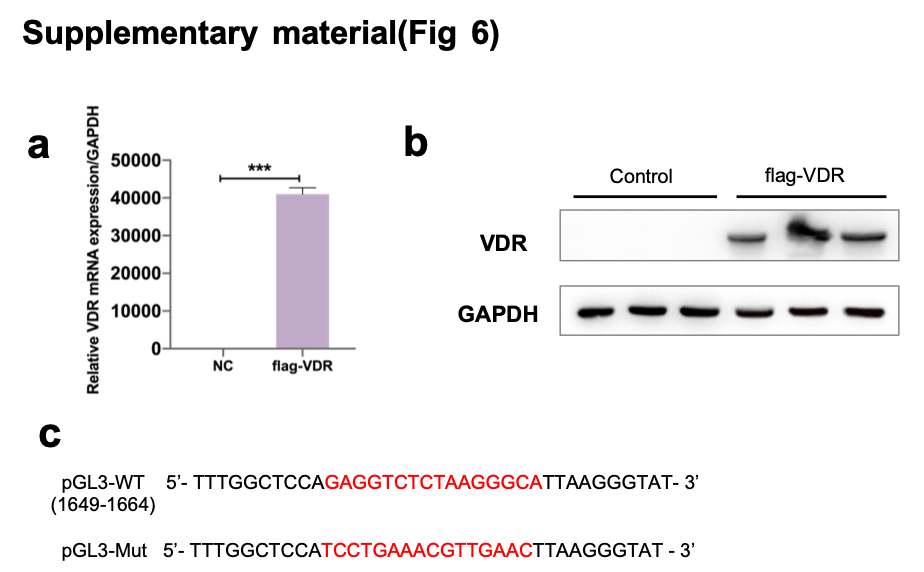

Supplement: Supplementary Figure 6 — RT-qPCR (A) and Western blot (B) results revealing successful transfection of the VDR gene into HEK-293T cells. (C) The sequence of the luciferase reporter plasmid (pGL3) harboring the CAMP promoter region with the WT sequence or the mutant VDR binding site. [file SupplementaryFigure_6.png]
